# Supplementary figures and images for: Synthesis and Adsorption Performance of a Hierarchical Micro-Mesoporous Carbon for Toluene Removal under Ambient Conditions
Source: Materials (Basel). 2020 Feb 5;13(3):716. doi: 10.3390/ma13030716 (PMC7040770; doi:10.3390/ma13030716)

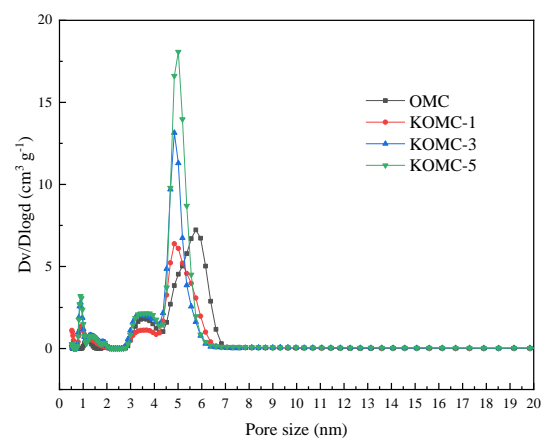

**Figure S1.** DFT pore size distributions

Supplement: Supplementary file 1 [file materials-13-00716-s001.pdf]
